# Detection of identical T cell clones in peritumoral pleural effusion and pneumonitis lesions in a cancer patient during immune-checkpoint blockade

**DOI:** 10.18632/oncotarget.25743

**Published:** 2018-07-17

**Authors:** Kentaro Tanaka, Toyoshi Yanagihara, Yuki Ikematsu, Hiroyuki Inoue, Keiichi Ota, Eiji Kashiwagi, Kunihiro Suzuki, Naoki Hamada, Ario Takeuchi, Katsunori Tatsugami, Masatoshi Eto, Kayo Ijichi, Yoshinao Oda, Kohei Otsubo, Yasuto Yoneshima, Eiji Iwama, Yoichi Nakanishi, Isamu Okamoto

**Affiliations:** ^1^ Research Institute for Diseases of The Chest, Graduate School of Medical Sciences, Kyushu University, Higashu-Ku, Fukuoka 812-8582, Japan; ^2^ Department of Urology, Graduate School of Medical Sciences, Kyushu University, Higashu-Ku, Fukuoka 812-8582, Japan; ^3^ Division of Pathophysiological and Experimental Pathology, Department of Pathology, Graduate School of Medical Sciences, Kyushu University, Higashu-Ku, Fukuoka 812-8582, Japan

**Keywords:** immune-related adverse event, immune-checkpoint inhibitor, bronchoalveolar lavage fluid, next-generation sequencing, complementarity-determining region

## Abstract

Although immune-related adverse events (irAEs) of treatment with immune-checkpoint inhibitors may be due to cellular immunity mediated by T lymphocytes, their pathogenesis has remained unknown. Here we collected bronchoalveolar lavage fluid (BALF) from a cancer patient with nivolumab-induced pneumonitis and isolated mononuclear cells for next-generation sequencing of the complementarity-determining region of the T cell receptor (TCR) β chain. Mononuclear cells in peritumoral pleural effusion isolated from the patient were similarly analyzed, and the results obtained for the two specimens were compared. A substantial number of TCRβ clones in BALF were also identified among lymphocytes in the peritumoral pleural effusion. Such a correlation was not apparent between TCRβ clones in BALF and those in peripheral blood. Moreover, many tumor-associated clones with a read frequency of ≥0.10% were also present in BALF. Our data suggest that irAEs might be induced by drug-activated lymphocytes originating from tumor tissue. Deep sequencing will thus be indispensable for investigations of the immune-based pathogenesis of, and the development of optimal treatments for, irAEs.

## INTRODUCTION

Immune-checkpoint inhibitors (ICIs) have shown marked efficacy in and improved the survival of individuals with various advanced cancers [1]. However, these drugs often trigger immune-related adverse events (irAEs) including pneumonitis. The presence of infiltrating T lymphocytes in organs affected by such irAEs has suggested that these events are due to cellular immunity mediated by T cells [2, 3, 4]. The immunologic mechanism responsible for irAEs has remained unclear, however, and their optimal treatment has yet to be established.

We have now performed next-generation sequencing of T cells in bronchoalveolar lavage fluid (BALF) collected from a patient with metastatic kidney cancer who developed pneumonitis during treatment with the ICI nivolumab. Analysis of T cell receptor (TCR) clonality revealed that tumor-infiltrating lymphocytes (TILs) may have contributed to pathogenesis of the irAE.

## RESULTS

**Figure 1:**
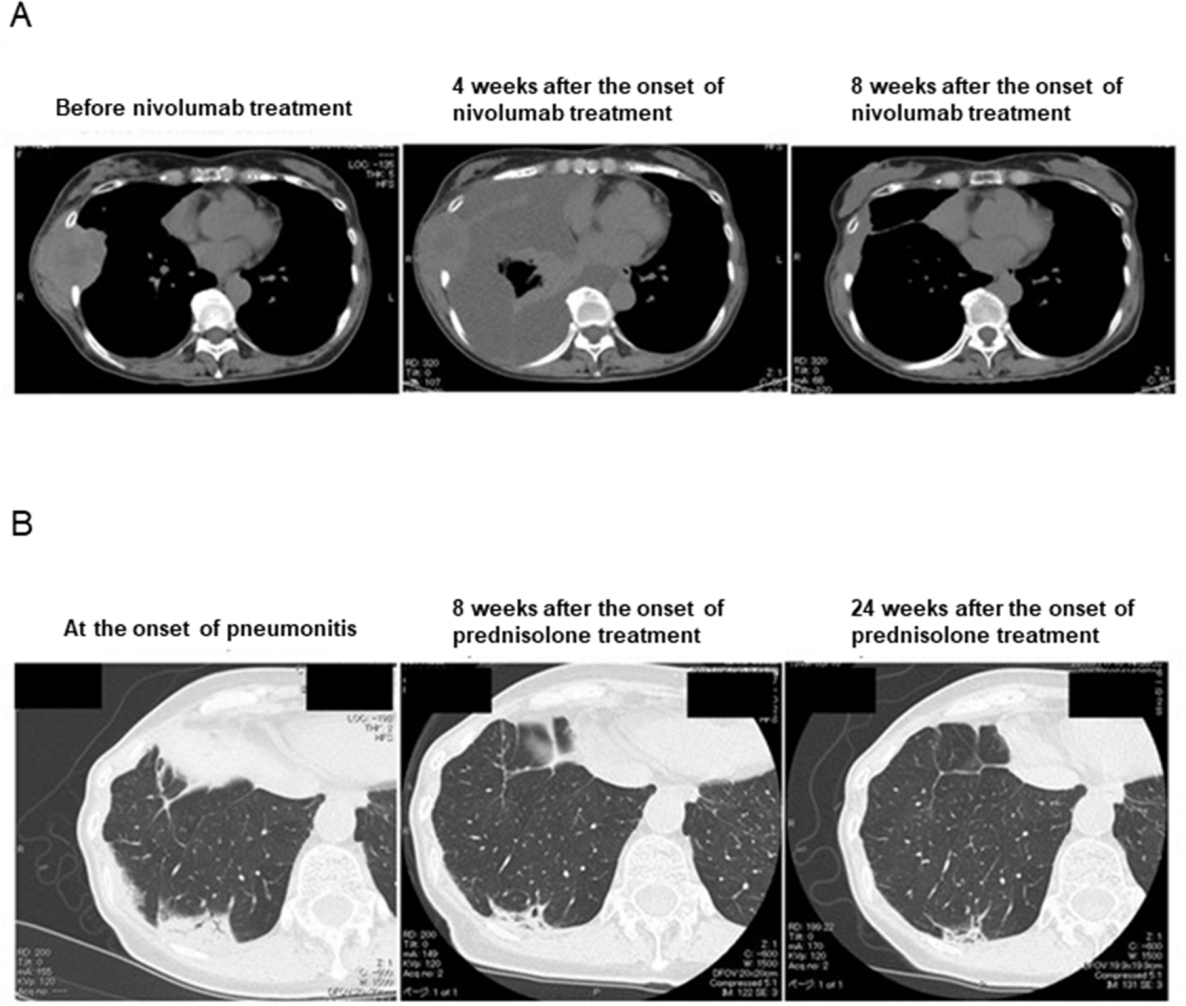
Chest CT scans of the patient **(A)** Clinical course of the pleural effusion and metastatic tumors. **(B)** Clinical course of the pneumonitis.

We previously described a patient with metastatic kidney cancer of the chest wall [5]. The patient was a 55-year-old woman with clear cell carcinoma who underwent radical nephrectomy (pT1bN0M0) in 2005. She developed new metastases in the right lung in October 2015, and these tumors were found to have invaded the thoracic wall in December 2016. Nivolumab therapy was then initiated. Four weeks after treatment onset, the patient developed massive right pleural effusion (Figure 1A). Thoracic drainage without pleurodesis resolved the effusion, and the metastatic tumors in the thoracic wall underwent marked shrinkage (Figure 1A). Cytological and flow cytometric analyses of the pleural fluid revealed that T lymphocytes were the predominant differentiated cell type, with no evidence of malignancy, and that the constituent CD8^+^ T cells expressed the immune-checkpoint proteins PD-1 (programmed cell death–1) and TIM-3 (T cell immunoglobulin domain– and mucin domain–containing protein–3). These observations suggested that the intrathoracic space reflected the peritumoral microenvironment and that the pleuritis was mediated by ICI-activated TILs that had exited from the tumor sites [6].

**Figure 2:**
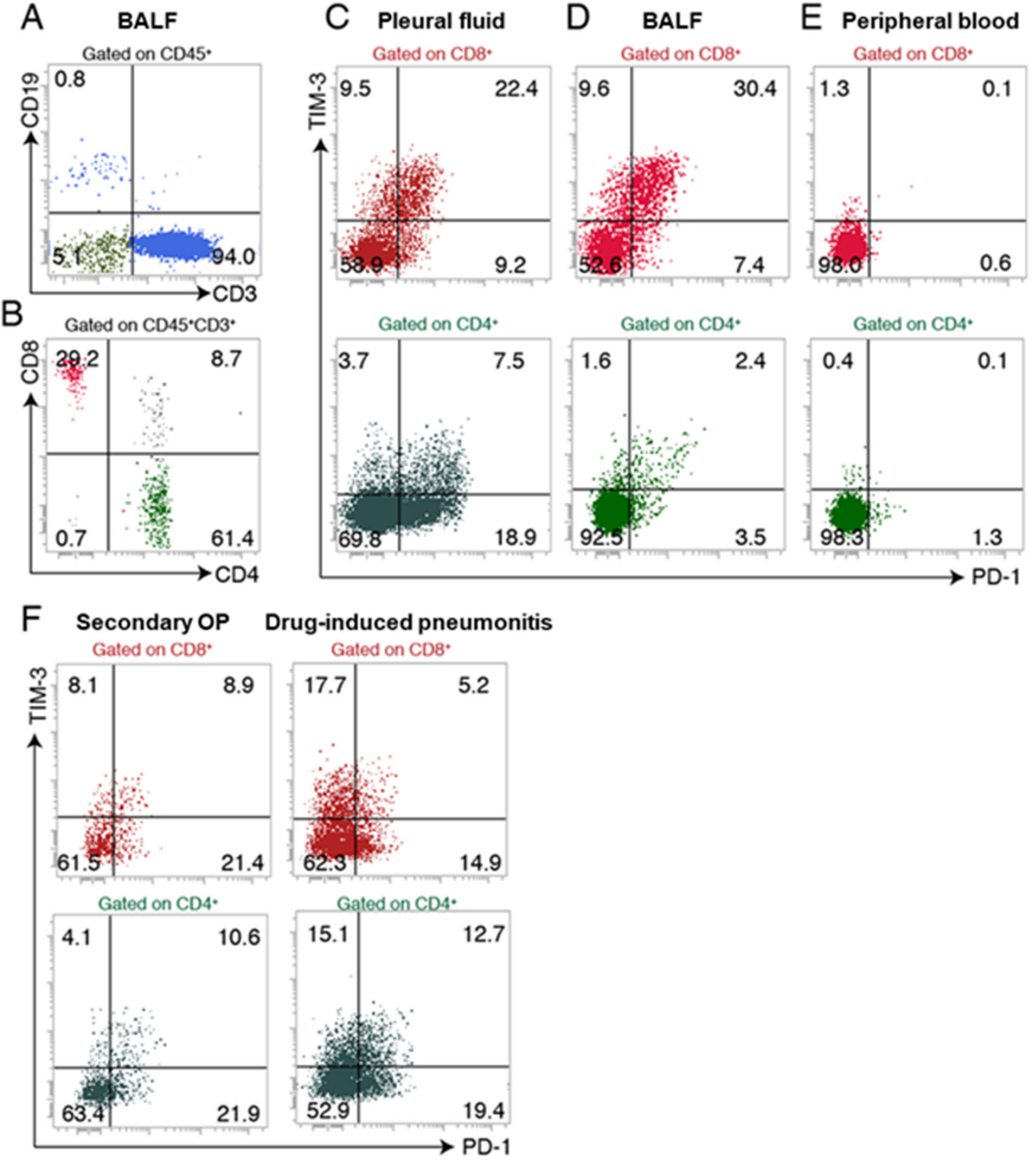
Flow cytometric analysis of lymphocyte subsets in BALF, pleural fluid, and peripheral blood of the patient **(A, B)** Expression of CD19 and CD3 on CD45^+^ cells (A) and expression of CD4 and CD8 on CD45^+^ CD3^+^ cells (B) in BALF. **(C–E)** Expression of PD-1 and TIM-3 on CD8^+^ T cells and CD4^+^ T cells in pleural fluid (C), BALF (D), and peripheral blood (E). **(F)** Expression of PD-1 and TIM-3 on CD8^+^ T cells and CD4^+^ T cells in BALF of patients with secondary organizing pneumonia (OP) or drug-induced pneumonitis.

The patient continued treatment with nivolumab after thoracic drainage. Four months after the initial onset of nivolumab treatment, chest computed tomography (CT) revealed nonsegmental subpleural and patchy consolidation in the bilateral lung field reminiscent of cryptogenic organizing pneumonia [7, 8, 9] (Figure 1B). Bronchoscopy was performed for collection of BALF from the consolidation area. Cytological analysis of the BALF revealed marked elevation of the lymphocyte fraction (43.6%), with 55.1% macrophages, 0.9% neutrophils, and 0.4% eosinophils and without any evidence of malignancy or infection. Flow cytometric analysis of the BALF revealed that 94.0% of the lymphocytes were CD3^+^ T cells (Figure 2A), with the CD4:CD8 ratio of these cells being 2.1:1 (Figure 2B). The expression pattern of PD-1 and TIM-3 on the surface of CD8^+^ T cells in the BALF was similar to that on those in the previously collected pleural fluid specimen (Figure 2C, 2D). In addition, this expression pattern differed markedly from that of these proteins on CD8^+^ T cells in BALF of patients with organizing pneumonia secondary to bacterial pneumonia or with cytotoxic drug–induced pneumonitis (Figure 2F). These findings of CT imaging, cytology, and flow cytometry indicated that the pneumonitis of the patient was induced by nivolumab-activated T lymphocytes. Nivolumab treatment was then discontinued and oral prednisolone was initiated at 0.5 mg/kg per day. Chest CT revealed subsequent amelioration of lung infiltration (Figure 1B).

**Figure 3:**
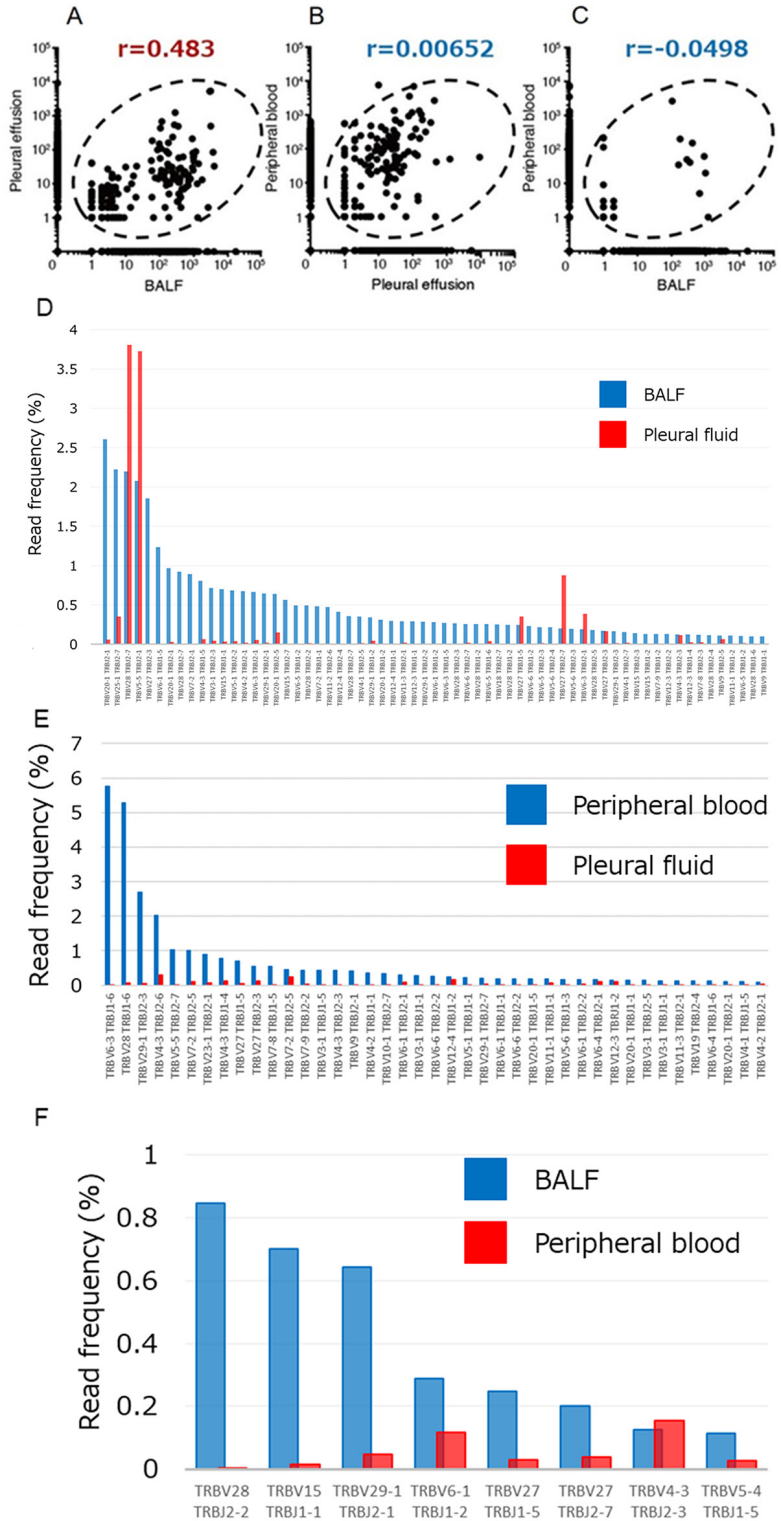
Relations among the numbers of TCRβ clones in pleural fluid, BALF, and peripheral blood of the patient **(A–C)** Numbers of clones in BALF and pleural effusion (A), in pleural effusion and peripheral blood (B), and in BALF and peripheral blood (C). Pearson’s correlation coefficient (r) values are shown. **(D)** T cell clones in BALF (read frequency of ≥0.1%) compared with those detected in pleural fluid. **(E)** T cell clones in peripheral blood (read frequency of ≥0.1%) compared with those detected in pleural fluid. **(F)** T cell clones in BALF (read frequency of ≥0.1%) compared with those detected in peripheral blood. X and Y axis indicate each clone of TCRβ and read frequency, respectively.

To characterize further the clonality of T cells in BALF and pleural fluid specimens of the proband, we performed next-generation sequencing for the complementarity-determining region (CDR) of the TCRβ chain. We obtained total sequence reads of 234,696 and 261,855 as well as in-frame reads of 143,292 and 141,928 for lymphocytes in BALF and pleural fluid, respectively. Many TCR clones with a read frequency of ≥0.05% were detected in both specimens, and the numbers of individual clones were substantially correlated between BALF and pleural fluid (r = 0.483) (Figure 3A), suggesting that T cells in the pneumonitis lesion were largely derived from nivolumab-activated lymphocytes. In addition, among CDR sequences detected in pleural fluid, >60 specific TCR clones with a read frequency of ≥0.10% were observed in BALF (Figure 3D). To exclude the possibility that expanded clones in peripheral blood might have contributed to the induction of pneumonitis, we also analyzed TIM-3 and PD-1 expression on T cells in peripheral blood as well as the relation of TCR clonality in peripheral blood to that in BALF or pleural effusion. T cells expressing PD-1 or TIM-3 were virtually undetectable in peripheral blood (Figure 2E), and the numbers of TCR clones in peripheral blood were not correlated with those in pleural effusion or BALF (r = 0.00652 and –0.0498, respectively) (Figure 3B, 3C). Although some clones detected in pleural fluid were also expanded in peripheral blood, the number of such common expanded clones was small (Figure 3E). In the case of BALF and peripheral blood, the few common clones detected were present at a relatively low frequency (Figure 3F).

## DISCUSSION

According to an official American Thoracic Society clinical practice guideline, cellular analysis of BALF and examination of a high-resolution CT scan are recommended to support a specific diagnosis of interstitial lung disease [10]. In the present study, we performed flow cytometric analysis of the immune-checkpoint proteins PD-1 and TIM-3 as well as next-generation sequencing for analysis of TCR clonality in T cells of BALF collected after the development of pneumonitis in a patient with metastatic kidney cancer that had invaded the chest wall. We detected a substantial frequency of CD8^+^ T cells positive for both PD-1 and TIM-3 in both BALF and pleural fluid. Moreover, even with a high cutoff threshold of 0.10% for read frequency, the concordance of individual TCR clones between these two specimens was marked. These findings thus support the notion that TCR clones in this patient may recognize antigens shared by the metastatic tumors of the thoracic wall and by the lung. Our study also shows that BALF is suitable for collection of appropriate T cells for deep sequencing. As far as we are aware, this is the first report of TCR clonality analysis in ICI-induced pneumonitis with the use of T cells in BALF, which may directly reflect the immunologic microenvironment of pneumonitis. Although many expanded clones were also detected in peripheral blood, little correlation was apparent between these clones and those in BALF. We therefore conclude that the pneumonitis of the patient was induced by nivolumab-activated peritumoral lymphocytes originating from the pleural space.

Two recent studies examined TCR clonality to determine the relation between TILs and T cells associated with irAE lesions in patients treated with ICIs. The first study described two patients treated with ipilimumab and nivolumab and found identical clones of T cells associated with the tumor and the organ affected by the irAE (cardiac muscle) [3]. The second study described a patient with pneumonitis whose tumor showed a response to pembrolizumab, with comparison of the TCR repertoire between the primary tumor and resected pneumonitis lesion revealing many overlapping clones [4]. We are not able to confirm definitively that the pneumonitis of the present patient was caused by nivolumab-activated TILs because we did not compare T cell clonality between lymphocytes isolated from tumor tissue and those in the pleural fluid. This is a limitation of our study and is attributable to the fact that biopsy of the thoracic tumors was not appropriate clinically.

Several recent studies that examined the association of a tumor response with irAEs have suggested that the occurrence of irAEs including pneumonitis may be a biomarker of nivolumab efficacy [11, 12, 13, 14]. It is therefore possible that the response to PD-1–specific antibodies such as nivolumab is related to the number of expanded T cell clones, which in turn is related to the frequency of irAEs in various organs. In the present patient, a sustained tumor response and rapid resolution of pneumonitis were apparent after the cessation of nivolumab treatment and the onset of oral corticosteroid administration. The question then arises as to why common clones of T cells in the tumor and irAE lesions respond differently to immunosuppression. Differences in the immune microenvironment between the tumor mass and the tissue affected by the irAE might result in differences in the reactivity or affinity of the expanded T cell clones for the recognized antigens. Prospective studies will be necessary to elucidate the complex relations among the type of ICI, the clonality of T cells infiltrating tumors and irAE lesions, and the type of immunosuppression.

Although only one patient was analyzed and the concordance of T cell clones between BALF and the pneumonitis tissue was not demonstrated, we detected commonly expanded T cell clones in two spatially distinct compartments—the pleural cavity and lung—in a patient treated with nivolumab. Our findings support the notion that the ICI-induced immune response manifested through reaction of such T cells with shared antigens in the tumor and normal lung tissue. Furthermore, they indicate that next-generation sequencing not only contributes to an understanding of the pathogenesis of irAEs in patients treated with ICIs but also informs the development of appropriate treatments.

## MATERIALS AND METHODS

### BALF collection

BALF and peripheral blood were collected at the time the CT scan revealed lung consolidation at 4 months after the onset of nivolumab treatment. For BALF collection, we inserted a bronchofiberscope into segment 8 of the right lower lobe with the patient in the lateral position. The total cell count of the specimen was 19.5 × 10^4^/ml, with a recovery rate of 58.7% (88/150 ml) and lymphocytes accounting for 43.6% of mononuclear cells.

### Flow cytometry

Flow cytometry was performed with Brilliant Blue 515–conjugated antibodies to human CD3 (UCHT1), peridinin chlorophyll protein complex (PerCP)– and cyanine 5.5 (Cy5.5)–conjugated antibodies to human CD4 (RPA-T4), and phycoerythrin (PE)– and Cy7-conjugated antibodies to human CD8 from BD Bioscience, as well as with PerCP- and Cy5.5-conjugated antibodies to human CD19 (HIB19), allophycocyanin (APC)–conjugated antibodies to human CD45 (HI30), PE-conjugated antibodies to human PD-1 (CD279, MIH4), PE-conjugated mouse immunoglobulin G1 κ isotype control (MOPC-21), APC-conjugated antibodies to human TIM-3 (CD366, F38-2E2), and APC-conjugated mouse immunoglobulin G1 κ isotype control (MOPC-21) from Biolegend. Cells were incubated for 10 min on ice with Human BD Fc Block (0.5 μg/ml, BD Bioscience) to block Fc receptors before exposure to antibodies for 20 min on ice and subsequent analysis with a FACS Verse instrument (BD Bioscience).

### Human TCR repertoire analysis by next-generation sequencing

Next-generation sequencing analysis was performed with an unbiased TCR repertoire analysis technology developed by Repertoire Genesis (Osaka, Japan). In brief, after obtaining written informed consent, we collected pleural effusion, BALF, and whole blood from the patient. Mononuclear cells were isolated by density gradient centrifugation with Ficoll-Paque PLUS (GE Healthcare Health Sciences), and total RNA was extracted from the cells and purified with the use of an RNeasy Mini Kit (Qiagen). The RNA was converted to cDNA with the primer BSL-18E (5'-AAAGCGGCCGCATGCTTTTTTTTTTTTTTTTTT-3') and Superscript III reverse transcriptase (Invitrogen), after which double-stranded cDNA was synthesized with *Escherichia coli* DNA polymerase I, *E. coli* DNA ligase, and RNase H (Invitrogen–Thermo Fisher Scientific), was rendered blunt-ended with T4 DNA polymerase (Invitrogen–Thermo Fisher Scientific), was ligated at the 5' end to P20EA (5'-TAATACGACTCCGAATTCCC-3') and P10EA (5'-GGGAATTCGG-3') adapters, and was then cut with the NotI restriction enzyme. The cDNA was then subjected to the polymerase chain reaction (PCR) with KAPA HiFi DNA Polymerase (Kapa Biosystems) and both a TCRβ-chain constant region–specific primer (CB1, 5'-GAACTGGACTTGACAGCGGAACT-3') and P20EA. The PCR protocol included 20 cycles of incubations at 98°C for 20 s, 65°C for 30 s, and 72°C for 1 min. A second PCR was performed with CB2 (5'-AGGCAGTATCTGGAGTCATTGAG-3') and P20EA under the same conditions. The second set of PCR products was then amplified with P22EA-ST1-R (5'-GTCTCGTGGGCTCGGAGATGTGTATAAGAGACAGCTAATACGACTCCGAATTCCC-3') and CB-ST1-R (5'-TCGTCGGCAGCGTCAGATGTGTATAAGAGACAGGCTCAAACACAGCGACCTC-3'), and index (barcode) sequences were added to the amlicons with the use of a Nextera XT index kit v2 setA (Illumina). The indexed amplicon products were mixed in equal molar concentrations and quantified with a Qubit 2.0 Fluorometer (Thermo Fisher Scientific). Sequencing was performed with the Illumina Miseq paired-end platform (2 × 300 bp). Assignment of sequences was performed by determination of sequences with the highest identity in a data set of reference sequences from the International ImMunoGeneTics (IMGT) database (http://www.imgt.org). Data processing, assignment, and data aggregation were automatically performed with software developed in-house by Repertoire Genesis. CDR3 regions from a conserved cysteine at position 104 (Cys^104^) to a conserved phenylalanine at position 118 (Phe^118^) and the following glycine (Gly^119^) were determined. The copy numbers of identical unique reads were automatically counted, and the percentage frequency of each unique read was calculated.
